# Suicidal Behaviors among Adolescents in Juvenile Detention: Role of Adverse Life Experiences

**DOI:** 10.1371/journal.pone.0089408

**Published:** 2014-02-24

**Authors:** Madhav P. Bhatta, Eric Jefferis, Angela Kavadas, Sonia A. Alemagno, Peggy Shaffer-King

**Affiliations:** 1 Department of Biostatistics, Environmental Health Sciences and Epidemiology, College of Public Health, Kent State University, Kent, Ohio, United States of America; 2 Department of Social and Behavioral Sciences, College of Public Health, Kent State University, Kent, Ohio, United States of America; 3 Department of Health Policy and Management, College of Public Health, Kent State University, Kent, Ohio, United States of America; Pennsylvania State University, United States of America

## Abstract

**Purpose:**

The purpose of this study was to assess the influence of multiple adverse life experiences (sexual abuse, homelessness, running away, and substance abuse in the family) on suicide ideation and suicide attempt among adolescents at an urban juvenile detention facility in the United States.

**Materials and Methods:**

The study sample included a total of 3,156 adolescents processed at a juvenile detention facility in an urban area in Ohio between 2003 and 2007. The participants, interacting anonymously with a voice enabled computer, self-administered a questionnaire with 100 items related to health risk behaviors.

**Results:**

Overall 19.0% reported ever having thought about suicide (suicide ideation) and 11.9% reported ever having attempted suicide (suicide attempt). In the multivariable logistic regression analysis those reporting sexual abuse (Odds Ratio = 2.75; 95% confidence interval  = 2.08–3.63) and homelessness (1.51; 1.17–1.94) were associated with increased odds of suicide ideation, while sexual abuse (3.01; 2.22–4.08), homelessness (1.49; 1.12–1.98), and running away from home (1.38; 1.06–1.81) were associated with increased odds of a suicide attempt. Those experiencing all four adverse events were 7.81 times more likely (2.41–25.37) to report having ever attempted suicide than those who experienced none of the adverse events.

**Conclusions:**

Considering the high prevalence of adverse life experiences and their association with suicidal behaviors in detained adolescents, these factors should not only be included in the suicide screening tools at the intake and during detention, but should also be used for the intervention programming for suicide prevention.

## Introduction

In 2010, 1,926 adolescents aged 10–19 years died as a result of suicide, making it the third leading cause of death among adolescents in the United States (U.S.) [1]. For every completed suicide many more adolescents seriously think about attempting suicide (*suicide ideation*), make a plan to attempt suicide (*suicide plan*), and actually attempt suicide one or more times (*suicide attempt*) [2]. In addition, there are non-fatal attempts that result in an injury that requires medical attention. Therefore, completed suicides are only a fraction of the larger public health concern of suicide and suicidal behaviors among adolescents.

Suicide is the leading cause of death among adolescents in juvenile detention facilities [3], with rates 3 to 18 times higher than the age-matched general population adolescents [4]. Similarly, rates of suicidal behaviors (ideation, plan and attempt) also tend to be higher among incarcerated adolescents than general population adolescents [5], [6]. Among U.S. adolescents in juvenile detention facilities, the estimates of the lifetime prevalence of suicide ideation range from 22.0% to 58.3% and of a lifetime suicide attempt range from 11.0% to 33.0% [5], [7], [8], [9], [10]. Adolescent suicide and suicidal behaviors, therefore, are of special interest for the juvenile detainee population.

Risk factors for suicide and suicidal behaviors in incarcerated adolescents include: mental disorders (borderline personality disorder traits; affective disorders; substance use disorders; post-traumatic stress disorder; anxiety; social phobia; and attention-deficit hyperactivity disorder) [3],[5], [7], [11],[12], [13], [14], [15], [16], [17], [18, [19]; female gender [5], [6], [8]; race [11], [20]; impulsivity [10], [13]; anger [14], [17]; a tendency to act out [18], [21]; younger age [5]; and perceived negative parenting [16]. Adolescents in juvenile detention often have higher rates of risk factors for suicide including substance abuse, mental disorders, trauma, and stressful life events than the general population youths [22]. Moreover, being in juvenile detention is itself a highly stressful event that may increase the risk of suicide among these adolescents. Therefore, screening at *intake* to a juvenile justice facility to identify individuals at a high risk of suicide is important for suicide prevention among these vulnerable youths [23].

Only a limited number of studies have assessed the role of adverse life experiences on suicidal behaviors in incarcerated adolescents. In previous studies, sexual abuse, exposure to violence, and housing stress have been reported as potential risk factors for suicide ideation and attempt among incarcerated adolescents [5], [8], [9], [24], [25], [26]. However, major limitations of these studies include the limited adverse life experiences studied and the small sample sizes, especially for females. To our knowledge, this is one of the largest epidemiologic studies to assess the influence of multiple adverse life experiences (sexual abuse, homelessness, running away, and substance abuse in the family) on suicide ideation and suicide attempt among male and female adolescents at an urban juvenile detention facility in the U.S. Understanding the influence of various risk factors on suicide ideation and attempt among adolescents in juvenile justice facilities has implications for developing appropriate screening tools and intervention programs aimed at suicide prevention.

## Materials and Methods

### Ethics Statement

This was a voluntary and anonymous survey and sex was the only identifiable demographic information collected. To maintain total anonymity of the participants, requirement for written informed consent/assent was waived. The waiver of written consent/assent and the overall study was approved by the University of Akron Institutional Review Board.

### Participants

Participants in the study were adolescents processed at a juvenile detention facility in an urban area in Ohio. The study population and methods for data collection have been previously described [27]. Briefly, during the intake process at the facility between 2003 and 2007, adolescents were asked to participate in a voluntary, anonymous survey of risk and protective factors for adverse health outcomes. Seventy-six percent of the adolescents entering the facility were male. The racial/ethnic distribution of the population included 75.2% African Americans, 20.7% whites, 3.9% Hispanics, 0.1% Asians, and 0.1% others. The age distribution included: 2.6% younger than 12 years, 3.7% aged 12, 7.7% aged 13, 12.8% aged 14, 19.7% aged 15, 25.0% aged 16, and 28.3% aged 17 years. The detention center served a county with a population of 1.3 million and housed both pre- and post-adjudicated youths with various alleged offenses including personal, property, drug, public order, and unruly offenses. The average length of stay in the facility was 12.5 days. A total of 3,156 adolescents agreed to participate and completed all the key questions for the present analysis. The study sample accounted for 80% of all intakes at the detention center during the data collection period.

### Survey Instrument Administration

Adolescents self-administered the survey instrument by interacting anonymously with a voice enabled computer that read a set of 100 “yes” or “no” questions related to risk and protective factors that were heard over headphones. Adolescents responded by pressing “Y” or “N” on the computer keyboard. The instrument measured risk in the following domains: problems with alcohol and drug use; alcohol and drug treatment history; mental and physical health problems and treatment history for each; sexual behavior; anger management and physical violence; adverse life experiences; and lack of family support.

### Primary Outcomes of Interest

The primary outcomes of interest in this study were *suicide ideation* and *suicide attempt. Suicide ideation*, defined as ever having thoughts about killing oneself, was measured using a “Yes” or “No” response to the question *“Have you ever thought about killing yourself?”. Suicide attempt*, defined as ever having tried to kill oneself, was measured using a “Yes” or “No” response to the question “*Have you ever tried to kill yourself?”.*

### Primary Exposures of Interest

#### Adverse life experiences

The four *adverse life experiences* measured included: sexual abuse (*anyone had ever touched them or done anything to them in a sexual way that they did not want to have done*); drug or alcohol abuse by a member of the household (*anyone in their home drink or use drugs enough to embarrass or upset them*); running away from home (*had ever stayed away from home for more than two nights because they didn't want their family to know about something or they were afraid to go home*), and homelessness (*had ever lived on the street or in a shelter*). The independent and joint effects of each adverse life experience with suicide ideation and suicide attempt were assessed.

### Secondary Exposures of Interest

#### Other individual and family related risk factors

Physical health symptoms were measured using six items that asked the adolescents of any of the following symptoms currently: night sweats, diarrhea, swollen glands, loss of weight without intention, coughing blood, or shortness of breath. Recent medical problems were measured using the following three items: had experienced any serious medical problems in the past year; currently under the care of a physician; or taking medication for a health problem. Family support was measured using three items: when something went wrong, their family was there to help them; they could depend on their family; and their family helped them to be the person that they wanted to be. Risky sexual behavior was measured by four items: first sexual experience younger than age 13; ever had unprotected sex; sex while drunk or high; and ever having exchanged sex for anything (e.g., drugs, money).

#### Substance abuse risk factors

Drug use, measured by eight items, addressed the use of drugs at least once a month during the preceding six months: used [marijuana; powder or crack cocaine; LSD (acid); heroin; inhalants (huffing or sniffing things like glue, paint thinner, gasoline, etc.); uppers (speed, amphetamines, meth, crystal meth, crank); downers (valium, librium, Xanax, barbs, barbiturates); or other substances] at least once a month for the past 6 months. Problem with alcohol use was measured using eight items: drank more than one drink of alcohol just about every day; when drinking, drank four or more drinks during that day; drinking ever kept them from doing things they were supposed to do; was hard to stop drinking once they started; ever wanted to keep drinking after their friends have had enough; ever drank secretly or when alone; ever gotten into a fight when drinking; and people nagged them or complained about their drinking. History of substance abuse treatment was measured using two items: ever gone to treatment, a counselor, or a doctor because of alcohol use; and ever gone to treatment, a counselor, or a doctor because of drugs use.

#### Mental health risk factors

*History of mental health treatment* was measured using four items: *had ever seen or were currently seeing a counselor or psychologist because of school, family or personal problems*; *had ever been hospitalized for mental health or emotional problems*; and *taking prescription medication for mental health problems*. *Symptoms of depression* was measured using six items: *lost interest in things that they usually did at home, at school or with their friends*; *felt sad or tired most of the time*; *felt like their life was a mess and will never get better; felt like they are unimportant or worthless*; *hard to concentrate at school or work; and thought about death a lot. Anger management issue* was measured using four items: *had a bad temper that they couldn't control sometimes*; *got into physical fights at school or at home that they regretted later*; *have had arguments at home where they threatened to hurt each other*; and *have had physical fights in their home where they hurt each other*.

For each of the secondary exposure of interest, an index score was created for the variable by summing the response to each question in the category. The score was dichotomized as an adolescent reporting no history of exposure (score  = 0) or any history of exposure (score ≥1) for that particular variable.

### Statistical Analyses

The differences in the outcomes and exposure variables of interest between males and females were assessed using the χ^2^-test. To assess the individual relationship of adverse life experiences and other exposures/potential risk factors with suicide ideation and suicide planning, univariable logistic regression analyses were performed and crude odds ratio (OR) and the associated 95% confidence intervals (CI) are reported. Multivariable logistic regression analyses were performed to assess the independent effect of the primary exposure variables with suicide ideation and suicide attempt, respectively, while controlling for the potential confounders. The final logistic regression models included all the variables significantly associated with the outcomes at α = 0.05 in the univariable analyses. The combined effects of multiple adverse life experiences were computed by exponentiation of the sum of logistic regression coefficient for independent effects. Multivariable logistic regression analyses stratified by gender were performed and the OR and associated 95% CI are reported. All the data analyses were performed using SAS® 9.3.1 (SAS Institute, Cary, NC).

## Results

Of the total 3,156 adolescents participating in the study, 694 (22.0%) were females and 2,462 (78.0%) were males. Overall, 42.7% of the adolescents reported at least one of the four adverse life experiences, with 24.5%, 12.7%, and 5.5% respectively reporting 1, 2, and 3 or 4 adverse life experiences. **Table 1** presents the overall and sex stratified prevalence of specific adverse life experience and other potential risk factors for suicide ideation and attempt among the adolescents in the study. Females were significantly more likely to report experiencing each of the four adverse life events than males (p<0.0001). Females also significantly reported higher levels of risky sexual behaviors, experiencing physical health symptoms, receiving medical care for a health problem in the past year, and mental health related problems than males. Additionally, females reported a significantly lower level of family support than males. There was no significant difference in the substance abuse risk factors between male and female adolescents in this study.

**Table 1:** Suicidal Behaviors and Potential Risk Factors by Sex Among Adolescents in an Urban Juvenile Detention Center in Ohio, 2003–2007.

	All (N = 3,156)	Male (n = 2,462)	Female (n = 694)	
	Proportion(95% CI)*	Proportion(95% CI)*	Proportion(95% CI)*	*p-value^¶^*
*Suicide Ideation and Attempt (Yes)*				
Ever thought about suicide	19.0 (17.7–20.4)	14.1 (12.7–15.5)	36.6 (33.0–40.2)	<.0001
Ever attempted suicide	11.9 (10.8–13.1)	8.1 (7.0–9.2)	25.4 (22.2–28.6)	<.0001
*Adverse Life Experiences*				
Any	42.7 (41.0–44.5)	36.1 (30.3–38.1)	66.0 (62.5–69.5)	<.0001
Sexual abuse	12.2 (11.1–13.3)	4.6 (3.7–5.4)	39.5 (35.8–43.1)	
Homelessness	16.9 (15.6–18.2)	15.4 (14.0–16.9)	21.9 (18.2–25.0)	
Running away from home	26.0 (24.5–27.6)	21.5 (19.9–23.1)	42.1 (38.4–45.8)	
Drug use in the family	12.6 (11.4–13.7)	11.2 (10.0–12.5)	17.3 (14.5–20.1)	
*Other Individual and Family Related Factors (Index score ≥1)* ^§^				
Risky sexual behaviors	74.5 (72.9–76.1)	73.2 (71.4–74.9)	79.1 (76.1–82.1)	0.0016
Physical health symptoms	33.8 (32.2–35.5)	30.0 (28.2–31.8)	47.4 (43.7–51.1)	<0.001
Recent medical problems	43.9 (42.2–45.7)	40.3 (38.5–42.3)	56.9 (53.1–60.6)	<.0001
Family support	89.1 (88.0–90.2)	90.8 (89.6–91.9)	83.1 (80.4–85.9)	<.0001
*Substance Abuse Factors (Index score ≥1)* ^§^				
Drug use in the prior 6 months	40.0 (39.2–38.2)	39.7 (37.7–41.6)	40.8 (37.1–44.5)	0.6030
History of substance abuse treatment	17.5 (16.2–18.9)	17.5 (16.0–19.2)	17.6 (14.8–20.4)	0.9445
Problems with alcohol use	33.9 (32.3–35.6)	33.6 (31.8–35.5)	35.0 (31.5–38.6)	0.4967
*Mental Health Factors (Index score ≥1)* ^§^				
Symptoms of depression	69.3 (67.7–70.9)	67.3 (65.4–69.1)	76.7 (73.5–79.8)	<.0001
Problem with anger management	71.9 (70.5–73.4)	69.1 (67.2–70.9)	81.7 (78.8–84.6)	<.0001
History of mental health treatment	62.2 (60.5–63.9)	57.8 (55.8–59.7)	78.1 (75.0–81.2)	<.0001

^*^95% Confidence Interval *^¶^*χ^2^-Test; 2-sided *p-value* ^§^Index score range: Risky sexual behaviors: 0–4; Physical health symptoms: 0–7; Medical care: 0–3; Family support: 0–3; Drug use in the prior 6 months: 0–8; Problems with substance use: 0–4; History of substance abuse treatment: 0–2; Problems with alcohol use: 0–8; Depression: 0–6; Problem with anger management: 0–4; History of mental health treatment: 0–6.

### Suicide Ideation and Suicide Attempt

Overall 601 (19.0%) of the adolescents reported suicide ideation and 376 (11.9%) reported suicide attempt. Female adolescents were significantly more likely to report suicide ideation (36.6% *versus* 14.1%; p<0.001) and suicide attempt (25.4% *versus* 8.1%; p<0.001) than males (**Table 1** **)**.

**Table 2** presents the unadjusted and adjusted odds of suicide ideation and suicide attempt for adverse life experiences and other risk factors. In the multivariable logistic regression analyses, experiencing sexual abuse (OR = 2.75; 95% C.I.  = 2.08–3.63) and homelessness (1.51; 1.17–1.94) were associated with an increased odds of suicide ideation, while sexual abuse (3.01; 2.22–4.08), homelessness (1.49; 1.12–1.98), and running away from home (1.38; 1.06–1.81) were associated with increased odds of suicide attempt among adolescents. In addition, other factors significantly associated with both suicide ideation and suicide attempt included: being female; having physical health symptoms; having had or receiving medical care for a serious health problem in the past year; problem with alcohol abuse; having problems with anger management; having any symptoms of depression; and a history of mental health treatment (**Table 2**).

**Table 2:** Logistic Regression Analyses of Risk Factors for Suicide Ideation and Suicide Attempt Among Adolescents in an Urban Juvenile Detention Center in Ohio, 2003–2007.

Risk Factor	Suicide Ideation		Suicide Attempt	
	UnadjustedOR (95% CI)*	AdjustedOR (95% CI)*	UnadjustedOR (95% CI)*	AdjustedOR (95% CI)*
*Female*	3.50 (3.00–4.26)	1.72 (1.35–2.19)	3.85 (3.08–4.81)	1.65 (1.24–2.18)
*Adverse Life Experiences*				
Sexual abuse	6.70 (5.34–8.40)	2.75 (2.08–3.63)	7.62 (5.96–9.74)	3.01 (2.22–4.08)
Homelessness	2.83 (2.30–3.48)	1.51 (1.17–1.94)	3.04 (2.39–3.85)	1.49 (1.12–1.98)
Running away from home	3.08 (2.55–3.70)	1.20 (0.95–1.51)	3.61 (2.90–4.51)	1.38 (1.06–1.81)
Drug use in the family	1.94 (1.53–2.46)	1.05 (0.79–1.39)	2.32 (1.77–3.03)	1.26 (0.92–1.73)
*Other Individual and Family Related Factors (Index score ≥1 vs. 0)* §				
Risky sexual behaviors	1.34 (1.24–1.45)	1.00 (0.77–1.30)	1.41 (1.28–1.55)	0.99 (0.72–1.36)
Physical health symptoms	1.87 (1.70–2.05)	1.55 (1.26–1.92)	1.84 (1.66–2.02)	1.35 (1.05–1.75)
Recent medical problems	1.77 (1.62–1.95)	1.60 (1.30–1.98)	1.82 (1.63–2.03)	1.72 (1.33–2.22)
Family support	0.77 (0.71–0.83)	0.99 (0.73–1.35)	0.77 (0.70–0.85)	0.89 (0.63–1.26)
*Substance Abuse Factors (index score ≥1 vs. 0)* §				
Drug use in the prior 6 months	1.61 (1.43–1.82)	1.13 (0.90–1.41)	1.61 (1.41–1.83)	1.08 (0.83–1.42)
History of substance abuse treatment	1.52 (1.31–1.76)	0.99 (0.77–1.30)	1.59 (1.35–1.88)	1.10 (0.81–1.48)
Problem with alcohol use	1.27 (1.21–1.34)	1.43 (1.13–1.80)	1.27 (1.20–1.34)	1.46 (1.11–1.93)
*Mental Health Factors (index score ≥1 vs. 0)* §				
Symptoms of Depression	1.76 (1.67–1.86)	2.63 (1.93–3.58)	1.66 (1.56–1.76)	1.47 (1.03–2.09)
Problem with anger management	1.77 (1.65–1.90)	1.75 (1.30–2.35)	1.82 (1.67–1.98)	2.40 (1.59–3.61)
History of mental health treatment	2.56 (2.34–2.78)	3.50 (2.61–4.69)	2.80 (2.54–3.09)	4.68 (3.06–7.16)

^*^OR (95% CI): Odds Ratio (95% Confidence Interval).

§ Index score range: Risky sexual behaviors: 0–4; Physical health symptoms: 0–7; Medical care: 0–3; Family support: 0–3; Drug use in the prior 6 months: 0–8; Problems with substance use: 0–4; History of substance abuse treatment: 0–2; Problems with alcohol use: 0–8; Depression: 0–6; Problem with anger management: 0–4; History of mental health treatment: 0–6.

**Table 3** presents the results of the stratified analyses of the risk factors for suicide ideation and suicide attempt by sex. It is interesting to note that risky sexual behaviors was not associated with either suicide ideation (OR = 1.00) or suicide attempt (OR = 0.99) in the overall analyses. However, risky sexual behaviors was positively associated (2.19; 1.28–3.76) with suicide ideation among females and, while not statistically significant, negatively associated (0.77; 0.57–1.05) among males.

**Table 3:** Multivariable Logistic Regression Analyses by Sex of Risk Factors for Suicidal Ideation and Suicide Attempt Among Adolescents in an Urban Juvenile Detention Center in Ohio, 2003–2007.

Risk Factor	*Suicide Ideation*		*Suicide Attempt*	
	Male	Female	Male	Female
	AdjustedOR (95% CI)*	AdjustedOR (95% CI)*	AdjustedOR (95% CI)*	AdjustedOR (95% CI)*
*Adverse Life Experiences*				
Sexual abuse	3.09 (2.00–4.76)	2.43 (1.67–3.53)	4.03 (2.54–6.38)	2.39 (1.59–3.60)
Homelessness	1.55 (1.13–2.12)	1.51 (0.98–2.33)	1.37 (0.93–2.01)	1.63 (1.04–2.55)
Running away from home	1.25 (0.93–1.67)	1.05 (0.71–1.56)	1.43 (1.00–2.03)	1.39 (0.90–2.12)
Drug use in the family	1.20 (0.84–1.69)	0.85 (0.53–1.37)	1.31 (0.86–1.97)	1.21 (0.74–1.99)
*Other Individual and Family Related Factors (Index score ≥1 vs. 0)* §				
Risky sexual behaviors	0.77 (0.57–1.05)	2.19 (1.28–3.76)	0.94 (0.64–1.39)	1.29 (0.72–2.30)
Physical health symptoms	1.55 (1.20–2.02)	1.57 (1.07–2.28)	1.47 (1.06–2.04)	1.16 (0.76–1.77)
Recent medical problems	1.53 (1.18–1.98)	1.63 (1.12–2.38)	1.79 (1.28–2.50)	1.53 (1.01–2.32)
Family support	0.89 (0.60–1.33)	1.29 (0.79–2.10)	0.83 (0.52–1.34)	0.97 (0.58–1.61)
*Substance Abuse Factors (index score ≥1 vs. 0)* §				
Drug use in the prior 6 months	1.08 (0.82–1.42)	1.15 (0.77–1.73)	0.90 (0.64–1.27)	1.42 (0.91–2.21)
History of substance abuse treatment	1.02 (0.75–1.40)	0.87 (0.54–1.41)	0.98 (0.67–1.44)	1.36 (0.83–2.14)
Problem with alcohol use	1.28 (0.97–1.70)	1.88 (1.22–2.91)	1.54 (1.08–2.18)	1.33 (0.83–2.14)
*Mental Health Factors (index score ≥1 vs. 0)* §				
Symptoms of depression	2.98 (2.01–4.43)	2.08 (1.24–3.50)	1.72 (1.16–2.50)	1.11 (0.63–1.95)
Problem with anger management	1.95 (1.35–2.81)	1.44 (0.85–2.44)	2.37 (1.41–3.98)	2.52 (1.29–4.96)
History of mental health treatment	4.02 (2.81–5.75)	2.50 (1.48–4.23)	4.81 (2.85–8.11)	4.35 (2.08–9.12)

^*^OR (95% CI): Odds Ratio (95% Confidence Interval).

§ Index score range: Risky sexual behaviors: 0–4; Physical health symptoms: 0–7; Medical care: 0–3; Family support: 0–3; Drug use in the prior 6 months: 0–8; Problems with substance use: 0–4; History of substance abuse treatment: 0–2; Problems with alcohol use: 0–8; Symptoms of depression: 0–6; Problem with anger management: 0–4; History of mental health treatment: 0–6.

### Independent and Combined Effects of Adverse Life Experiences

**Table 4** presents the independent and combined effects of sexual abuse and other adverse experiences on the odds of suicide ideation and suicide attempt while controlling for other potential risk factors. Sexual abuse is the strongest adverse life experience predictor for both suicide ideation and suicide attempt. With an increasing number of adverse life experiences the odds of suicide attempts increased, indicating a positive dose-response relationship. An individual who experienced all four adverse events was 7.81 times more likely (95% C.I.: 2.41–25.37) to have ever attempted suicide than one who experienced none of the adverse events.

**Table 4:** Association of Multiple Adverse Life Experiences with Suicide Ideation and Suicide Attempt Among Adolescents in an Urban Juvenile Detention Center in Ohio, 2003–2007.

Risk Factors	*Suicide Ideation*	*Suicide Attempt*
	Adjusted§OR (95% CI)*	Adjusted§OR (95% CI)*
Sexual abuse only *versus* none	2.75 (2.08–3.63)	3.01 (2.22–4.08)
Sexual abuse and homelessness *versus* neither	4.54 (2.67–7.70)	4.48 (2.48–8.09)
Sexual abuse, homelessness, and running away from home *versus* none	5.45 (2.55–11.66)	6.19 (2.62–14.66)
Sexual abuse, homelessness, running away from home, and drug use in the family *versus* none	5.72 (2.02–16.21)	7.81 (2.41–25.37)

§ Adjusted for sex, family support, drug and alcohol use, sexual risk behaviors, physical health symptoms, recent medical problems, history of mental health treatment, symptoms of depression, and anger management problems.

^*^OR (95% CI): Odds Ratio (95% Confidence Interval).

## Discussion

The findings of this large epidemiologic study of suicide ideation and attempt among male and female adolescents in an urban U.S. juvenile detention center have implications for prevention, intervention, policy, and research. The present study adds to the existing knowledge about the risk factors for suicidal behaviors in male and female adolescents in juvenile detention facilities by assessing the independent and joint influence of multiple adverse life experiences. The key findings of this study were that a history of sexual abuse, homelessness, and running away from home were independently significantly associated with ever attempting suicide. In addition, we observed a dose-response relationship between the number of adverse life experiences and suicidal behaviors, particularly suicide attempt. An individual experiencing all four adverse events was 5.7 and 7.8 times more likely to report suicide ideation and suicide attempt, respectively, than someone experiencing none. Considering the substantial prevalence of adverse life experiences observed among adolescents in this study, these risk factors are likely to have significant impact on future suicidal behaviors in this population.

Adolescents entering the juvenile detention facility in this study were at a substantial and elevated risk of suicide ideation (19.0%) and suicide attempts (11.9%). These results are similar to a previous comparable study that reported a prevalence of 11.0% for ever attempted suicide in 1,829 adolescents surveyed at intake to a detention center in Chicago [8]. We also observed a significant difference in the prevalence of suicidal behaviors among males and females, which also is consistent with previous findings in both the general population and adolescents in the juvenile justice system [5], [6].

In terms of individual adverse life experiences, a history of sexual abuse was the strongest independent predictor for both suicide ideation (OR = 3.0) and suicide attempt (OR = 2.8), which is consistent with findings of a previous study of incarcerated adolescents in the U.S [5]. Sexual abuse, as operationalized in this study, encompasses a broad definition without specificity in terms of type, frequency, age at occurrence, duration, or the characteristics of the perpetrator [28]. Using this broad definition of this measure is likely to underestimate the true effect due to possible non-differential misclassification of the exposure. Therefore, the association between sexual abuse and suicide ideation and suicide attempt may be even stronger than the association observed in this study.

A history of sexual abuse has a stronger association in males with both suicide ideation (OR = 3.1 *vs.* 2.4) and suicide attempt (OR = 4.0 *vs.* 2.4) than females but the observed difference was not statistically significant. With a prevalence of 39.5%, females, however, were 8.5 times more likely than males to report a history of sexual abuse. This observed difference in the prevalence of sexual abuse between male and female adolescents is consistent with that observed in the general population of adolescents [29]. Moreover, female detainees in this study were also significantly more likely to report experiencing other adverse life experiences than males. Adverse life experiences, including sexual abuse, are likely to result in a greater impact on suicidal behaviors in females than males due to a higher level of exposure.

Significant gender differences in self-reported suicidal behaviors and adverse life experiences highlight the need for a gender specific screening tool and programming for suicide prevention among detained adolescents. It should be noted that while females generally report higher rates of suicide ideation and attempt, they typically have lower suicide completion rates [1], [6]. As such, rates of completed suicides should not be used in isolation to guide practice and policy because this single indicator would likely mask the seriousness of the issue in female detainees. The difference in the levels of adverse life experiences among males and females may partly explain the difference in suicidal behaviors by gender. Suicide screening tools at intake and during the period of detention, therefore, should include questions on the history of adverse life experiences to identify high-risk adolescents. Moreover, intervention programs should include methods to address the effects of adverse life experiences, especially in females.

We also observed a significant association of suicidal behaviors with currently experiencing poor physical health symptoms and a history of serious medical condition. To the best of our knowledge, no previous studies have examined these factors in incarcerated youth. However, in the general population of high school aged adolescents, those with physical disabilities or long-term health problems report higher risk of suicide ideation (OR = 2.7) and suicide attempt (OR = 3.2) than those without disabilities or health problems [30]. We also observed a statistically significant association of suicidal behaviors with mental health risk factors including self-reported history of any symptoms of depression, mental health treatment and anger management issues. These observations are consistent with previous studies which found an association between mental health issues and suicidal behaviors among adolescents in the juvenile justice system [11], [12], [13], [14], [15] [16], [17], [18, [19].

Although screening of all adolescents within the first 24 hours of intake at a facility reduces the odds of suicide attempts, the screening protocols for juvenile facilities vary in terms of adolescents screened, timing of screening, screening tools used, and the qualification of the screeners [23]. Routine suicide screening protocols for all adolescents would be a prudent practice at detention center intake and periodically during the period of detention. The findings of this study point to the need to take into consideration gender, the history of adverse life experience and current physical health status as well as mental health and anger management issues in a potential suicide screening tool, prevention and intervention efforts. Consequently, detention facilities should have the capacity to evaluate and monitor these risk factors, which is not always the case. Ideally, psychological screenings conducted at intake that point to an elevated risk of suicide would be followed up by appropriate gender specific intervention programming to reduce the risk of suicide among adolescents in the juvenile justice system.

This study has several strengths and limitations, thus the findings have to be interpreted accordingly. With a sample size of 3,156, it is one of the largest studies conducted among adolescents in juvenile detention to assess the influence of adverse life experiences in suicidal behaviors by gender. We were able to control for the potential confounding effects of many known risk factors for suicidal behaviors including substance abuse and a history of mental health problems to assess the independent effect of adverse life experiences. The Computer-Assisted anonymous self-administered questionnaire likely enabled the participants to respond to the questions more honestly, thus reducing information bias. Because we did not have information on the non-participants, we were unable to assess any potential differences between the study sample and the non-participants to evaluate selection bias. However, the high response rate likely minimized the selection bias, if any. Moreover, the study sample reflected the underlying population in terms of gender distribution, which indicates the overall representativeness of the study sample.

This study is based on cross-sectional data, thus, we simply do not know the temporal sequencing of the risk factors explored and the self-reported life-time suicidal behaviors. Clearly, it is important to study the timing of suicidal behaviors in the context of risk factors, especially in trying to establish a causal relation. However, for the purpose of screening to identify high-risk individuals, this may not be as important a limitation. Although the underlying population was predominantly African-American and aged 13–17 years, the information on the age and race/ethnicity of the individual participants was not collected. While limiting personal identifying information increased anonymity for participants and likely reduced information bias, we could not assess the confounding effect of these variables. Similarly, we were unable to assess the influence of other underlying risk factors such as socioeconomic status and sexual orientation in this study. Additionally, we did not assess impact of other adverse and traumatic life experiences such as peer bullying, physical abuse, family instability, and community violence. Further research should focus on assessing the influence of these adverse life experiences in order to gain a comprehensive understanding of their effect on suicidal behaviors on adolescents in juvenile detention.

Another limitation of the study is that we did not specifically measure various types of mental disorders, including depression, which have previously shown to be associated with suicidal behaviors [11], [12], [13], [14], [15] [16], [17], [18, [19]. Having any symptoms associated with depression, as defined in this study, is not a specific measure of depression. Therefore, this study did not assess the influence of specific mental health disorders including depression on suicidal behaviors. However, a history of having ever received treatment for mental health issues was measured in the study. Although the prevalence of having any symptoms of depression seems high (69%), the fact that 62% of the adolescents reported having ever received a mental health treatment points to a significant prevalence of mental health issues in this population. Finally, secondary risk factors assessed in this study may be mediators rather than being confounders in the association between adverse life experiences and suicidal behaviors [31]. Future studies should assess the potential mediation of the relationship, particularly by the history of mental health problems.

Understanding the risk factors for suicidal behaviors among detained adolescents is essential for intervention programs for suicide prevention within juvenile correctional facilities. The detained adolescents will eventually be released into the general public and continue to be at high risk for suicide. Therefore, health professionals within and outside the juvenile justice system who care for these adolescents must be aware of the impact of adverse life experiences on suicidal behaviors for effective therapy and intervention for suicide prevention in this high-risk population.
